# Deep Phylogenetic Divergence and Lack of Taxonomic Concordance in Species of *Astronotus* (Cichlidae)

**DOI:** 10.1155/2012/915265

**Published:** 2012-06-24

**Authors:** Olavo Pinhatti Colatreli, Natasha Verdasca Meliciano, Daniel Toffoli, Izeni Pires Farias, Tomas Hrbek

**Affiliations:** ^1^Laboratório de Evolução e Genética Animal (LEGAL), Universidade Federal do Amazonas (UFAM), 69077-000 Manaus, AM, Brazil; ^2^Departamento de Genética e Evolução, Universidade Federal de São Carlos (UFSCar), 18052-780 São Carlos, SP, Brazil

## Abstract

The neotropical cichlid genus *Astronotus* currently comprises two valid species: *A. ocellatus* Agassiz, 1831 and *A. crassipinnis* Heckel, 1840. The diagnosis is based on color pattern and meristics counts. However, body color pattern is highly variable between regions and the meristic counts show a considerable overlap between populations differing in color patterning. They do not represent true synapomorphies that diagnose species. Purportedly the only truly diagnostic character is the presence or absence of one or more ocelli at the base of the dorsal fin, diagnosing *A. ocellatus* and *A. crassipinnis*, respectively. Using the 5′ portion of the mitochondrial COI gene and EPIC nuclear markers, the validity of the dorsal ocelli as diagnostic character was tested in individuals sampled from ten localities in the Amazon basin. Analyses rejected the hypothesis that dorsal ocelli are diagnostic at the species level. However, they revealed the existence of five hypothetical, largely allopatrically distributed morphologically cryptic species. The phylogeographic structure is not necessarily surprising, since species of the genus *Astronotus* have sedentary and territorial habits with low dispersal potential. The distribution of these hypothetical species is coincident with patterns observed in other Amazonian aquatic fauna, suggesting the role of common historical processes in generating current biodiversity patterns.

## 1. Introduction

The neotropical cichlid genus *Astronotus* currently comprises two valid species: *A. ocellatus* Agassiz, 1831 and *A. crassipinnis* Heckel, 1840 [1]. Kullander [1] reports a number of diagnostic characters, however, with the exception of the presence of ocelli at the base of the dorsal fin in *A. ocellatus* and their absence in *A. crassipinnis*, all other characters show considerable overlap in their statistical distributions. The two species are characterized by differences in the modal number of lateral line scales (35 to 40 in *A. crassipinnis* versus. 33 to 39 in *A. ocellatus*), and the number of rays and spines of the dorsal fin (modal XIII.20 in *A. ocellatus* versus modal XII.21-22 in *A. crassipinnis*). There are also reported differences in color hue and patterning where *A. crassipinnis* is darker than *A. ocellatus*, the first light vertical bar is above the anal fin base in *A. ocellatus* versus. more anteriorly in *A. crassipinnis*, and *A. crassipinnis* has two more or less well-separated dark vertical bars in the position of the first light bar in *A. ocellatus*. Although proposed as diagnostic characters, the position of the vertical bars and body color appears highly variable between localities and individuals (authors' obs.), and the meristic counts are not truly diagnostic (are not synapomorphies) since they represent modal values and overlap between species.

While the presence of ocelli on the dorsal fin is considered a diagnostic character of *A. ocellatus*, Kullander ([1]; see http://www2.nrm.se/ve/pisces/acara/astronot.shtml), only individuals from Peru were analyzed by Kullander [1] in his reanalysis of the genus. Moreover, Kullander [1] raises the possibility that ocelli are unique to specimens of western Amazonia, requiring a possible reinstatement or reclassification of species considered synonyms of *A. ocellatus*. The geographic distribution of *A. ocellatus* spans the whole Amazon basin and the Oyapock and Approuague drainages. It does not include the Bolivian basin which is a subbasin of the Amazon basin.

The quantity and size of ocelli further appear to be influenced by reproductive state. In a study by Queiroz and Barcelos [2] of *Astronotus ocellatus* (diagnosed as such by the presence of ocelli) from the Mamirauá Sustainable Development Reserve located in the western Amazon north of the city of Tefé, the authors demonstrated that the number of ocelli and their size are positively and linearly correlated with gonadal development in both males and females. These potential difficulties do not prevent, however, the common acceptance of ocelli as strictly diagnostic character of the two species (e.g., [3]).

Of the type series of *A. crassipinnis*, only two syntypes from the Guaporé River are known. Other type material reported from the Negro and Branco Rivers according to Kullander [1] likely represents *A. ocellatus* or some undescribed species. *Astronotus crassipinnis* is therefore restricted to the upper Paraguay River and the Bolivian Amazon including the Guaporé, Mamoré, and Madre de Dios rivers. However, pending designation of a lectotype from the Guaporé River, Kullander [1] considers the classification of Paraguayan and Bolivian Amazonian specimens as *A. crassipinnis* provisory. Kullander [1] also recognizes that *A. ocellatus* could be restricted to the western Amazon and that *Astronotus ocellatus* var. *zebra* Pellegrin, 1904 and *Astronotus orbiculatus* Haseman, 1911 both described from Santarem and currently considered junior synonyms of *A. ocellatus* could represent valid species or may be synonyms of *A. crassipinnis*. Kullander [1, 4] further mentions the existence of an *Astronotus* species from the Orinoco basin but does not recommend any kind of classification of these specimens.

Phenotypic variation of *A. ocellatus* at the scale of the Amazon basin would not be surprising given the extent of geographic distribution of the species and the biology of cichlids. Both species of the genus *Astronotus* inhabiting lentic environments are sedentary. Males have strong territorial behavior, and both sexes build nests and exhibit parental care. First gonadal maturation occurs between 15 and 24 months, and reproduction may occur more than once a year. Both species are also relatively large for fishes of the family Cichlidae (up to 35 cm SL and 1.5 kg). The geographic distribution of species of *Astronotus* as well as the species themselves may therefore carry signatures of climatic and geological events.

While phenotypic variation is evident in the species of *Astronotus*, it is not clear if the currently used sets of characters are fully diagnostic. An alternative approach to species diagnosis may be through the use of DNA barcoding [5]. DNA barcoding has rapidly expanded in the last years, and already the fish faunas of several countries have been barcoded (e.g., [6–9]). One of the objectives of the DNA barcoding initiative is to generate a curated database of reference material. The usefulness of this database depends on the quality of the reference specimens and the quality of the underlying taxonomic information. For example, recently diverged species may share DNA barcodes (COI haplotypes), or multiple species may be subsumed within the same morphospecies, and both cases will lower the quality of the database. Identifying these instances is the first step in generating a reliable biodiversity database.

Many neotropical fish species have broad geographic distributions, often occurring allopatrically in the tributaries of the Amazon River, or are even shared between the Amazon and other South American basins (see [10]). While some species truly appear to be biological species with weak or nearly nonexistent population structuring across its distributional range (e.g., [11–14]), others probably comprise morphologically cryptic species complexes, recently diverged groups, or complexes of hybridizing groups (e.g., [15–18]).

The goal of this study was to assess population structuring and reassess the taxonomy of the genus *Astronotus *based on an analysis of molecular data and assess the utility of a traditionally used diagnostic character for the species *A. ocellatus* and *A. crassipinnis*.

## 2. Material and Methods

### 2.1. Sampling

Tissue samples (dorsal muscle or pectoral fins) were collected from specimens purchased directly from artisanal fishermen and from fishes sampled with 50 mm mesh gillnets. The tissues were deposited in the tissue collection of the Laboratory of Animal Genetics and Evolution, Federal University of Amazonas. Most individuals were photographed, and vouchers are being deposited at the ichthyological collection of the Instituto Nacional de Pesquisas da Amazonia (INPA).

**Figure 1:**
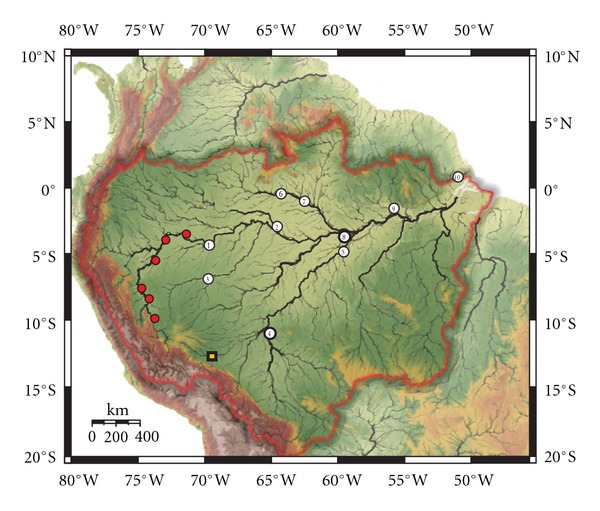
Sampling localities of species of *Astronotus* in the Brazilian Amazon. Base map was obtained from WWF (http://assets.panda.org/img/original/hydrosheds_amazon_large.jpg). Numbers correspond to sampling localities: (1) Tabatinga; (2) Mamirauá; (3) Juruá; (4) Guajará Mirim; (5) Borba; (6) Santa Isabel; (7) Barcelos; (8) Careiro Castanho; (9) Oriximiná; (10) Araguari. Red circles and yellow squares are localities of *A. ocellatus* and *A. crassipinnis*, respectively, studied by Kullander [1]. Reddish-brown line delimits the periphery of the Amazon basin.

**Figure 2:**
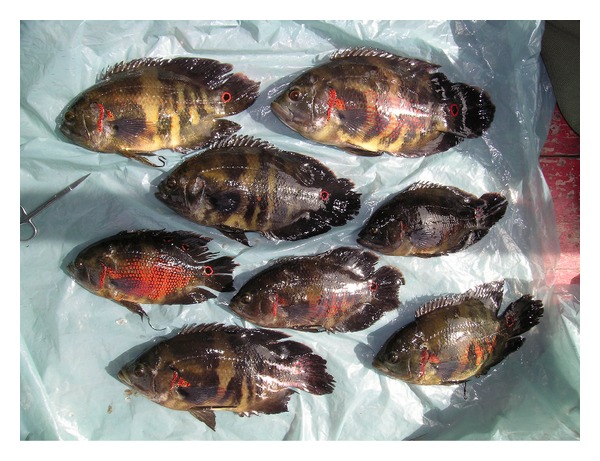
Photograph of fishes of the genus *Astronotus* collected in the Araguari River and showing the presence and absence of dorsal ocelli in the same locality. In addition to the Araguari locality, both *A. ocellatus* and *A. crassipinnis* phenotypes were collected in Oriximiná, Careiro Castanho, and Borba. Photo by S. C. Willis.

We sampled 10 localities in the Amazon basin (Figure 1), and individuals were classified as *A. ocellatus* or *A. crassipinnis* based on the presence/absence of at least one ocellus or dark spot on the posterior part of the dorsal fin (Table 1). We do not have exact information about the state of ocelli for the Tabatinga and Mamirauá/Tefé specimens; however, based on field identification, fishes from Tabatinga and Mamirauá/Tefé were classified as *A. ocellatus*. Several studies [2, 19] also only report *A. ocellatus* from Mamirauá. Similarly although the presence/absence of ocelli was not recorded for individual specimens at the time of collection at the localities of Careiro do Castanho and the Araguari River, both *A. ocellatus* and *A. crassipinnis* phenotypes were observed and sampled (Figure 2).

### 2.2. Polymerase Chain Reaction (PCR) and Sequencing

We amplified and sequenced one mitochondrial and two nuclear gene regions. All PCR reactions were carried out in a final volume of 15 *μ*L containing 7.0 *μ*L of ddH_2_O, 1.5 *μ*L of MgCl_2_ (25 mM), 1.5 *μ*L of dNTPs (10 mM), 1.2 *μ*L of 10x PCR buffer (100 mM Tris-HCl, 500 mM KCl), 1.2 *μ*L of each primer (2 *μ*M), 0.3 *μ*L of Taq DNA Polymerase (1 U/*μ*L), and 1 *μ*L of DNA (concentration varied between 50 ng and 100 ng). We amplified the COI barcode region with the primers COIFishF.2 (5′-CGACTAATCATAAAGATATCGGCAC-3′) and COIFishR.1 (5′-TTCAGGGTGACCGAAGAATCAGAA-3′), and the EPIC region primers 18049E2 (18049E2f2—5′-GTGGTGGAGATGCAYGAYGTGAC-3′; 18049E2r2—5′-TAGTAAAGGTCYCCRTGGATGGTGAG-3′), and 14867E4 (14867E4f2—5′- TGTGATCAGGGGACAGAGRAAAGGTG-3′; 14867E4r2—5′-CAGTARATGAACTGBCCGGTGTGG-3′) obtained from the online supplement of Li and Riethoven [20]. PCR reaction consisted of 35 cycles of denaturation at 93°C for 5 seconds, primer annealing at 50°C; 50°C and 56°C, respectively, for 35 seconds, and primer extension at 72°C for 90 seconds, followed by a final extension at 72°C for 5 minutes. PCR products were purified using the polyethylene glycol/ethanol precipitation [21] and subjected to cycle sequencing reaction using both amplification primers following the manufacturer's recommended protocol for BigDye sequencing chemistry (Applied Biosystems). Subsequent to the cycle sequencing reaction, the products were precipitated with 100% ethanol/125 mM EDTA solution, resuspended in Hi-Di formamide, and resolved on an ABI 3130xl automatic sequencer (Applied Biosystems). Base calls were verified by viewing electropherograms in the program Bioedit [22], sequences were aligned in the program Clustal W [23], and alignment was verified by eye. Sequences of nuclear genes were separated into alleles prior to analyses. Sequences were deposited in Genbank (JQ965997-JQ966020).

### 2.3. DNA Barcode Analysis (COI mtDNA)

Genetic distances between individuals were calculated using the JC69 model of molecular evolution [24], and individuals were clustered using the BIONJ algorithm [25]. The analyses were implemented in the online version of the ABGD software [26] whose objective is to automatically and in an unbiased way delimit clades. Clade delimitation was done assuming a range of possible intraclade **θ**s from 0.001 to 0.1. Once clades were identified, we also estimated average divergences between and within clades using the JC69 model of molecular evolution [24] in the program MEGA 5 [27]. Although the K2P model of molecular evolution [28] is the recommended [29] and has become the *defacto* model in DNA barcoding studies, it poorly fits the data at the species level divergence [30]. Collins et al. [30] recommend the use of uncorrected divergences or simplest models possible. Further, intraspecific divergences—employed in DNA barcoding threshold and barcoding gap methods, and pairwise divergences between sister taxa—employed in DNA barcoding gap methods, normally need no correction for multiple mutational hits and saturation due to their inherently shallow phylogenetic divergences.

We also performed an individual level Population Aggregation Analysis (PAA) [31] to identify clades. In the DNA barcoding literature, the use of molecular synapomorphies to delimit clades has been described by Rach et al. [32] under the acronym CAOS.

### 2.4. Phylogenetic Inference and Hypothesis Testing

Maximum likelihood topology for the mtDNA dataset was inferred in the program Treefinder [33], and the robustness of the tree topology was assessed using the nonparametric bootstrap with 1,000 replicates. The most appropriate model of molecular evolution for the mtDNA dataset was inferred as HKY85 [34] with a portion of the sites considered invariable in the program Treefinder [33]. Model selection criterion was the corrected Akaike Information Criterion [35]. Association of lineages and phenotypes was tested by comparing the constrained topology (phenotypes are monophyletic) with the most likely unconstrained topology. Significance was tested using the approximately unbiased test of Shimodaira [36]. A test of phylogenetic distribution of ocelli was performed using the CAPER package [37] in the statistical program R (http://www.cran.r-project.org/). A test of genetic structuring at nuclear loci, assuming the existence of groups identified in the ABGD [26] analysis of the COI barcode region, was performed in the software Arlequin 3.5.1 [38].

### 2.5. Phylogenetic Networks

Due to the low number of variable sites, phylogenetic relationships of nuclear haplotypes were inferred as a haplotype network using the PEGAS package [39] in the statistical program R (http://www.cran.r-project.org/).

## 3. Results

We sequence data for one mitochondrial and two nuclear DNA regions. We collected 664 bp of the mtDNA COI barcode region, representing 19 haplotypes separated by 31 mutations. No stop codons were observed in the COI barcode region. We also collected 397 bp of the nDNA 18049E2 EPIC regions, representing three haplotypes separated by three mutations. We further collected 248 bp of the nDNA 14867E4 EPIC region, resulting in two haplotypes separated by one mutation.

**Figure 3:**
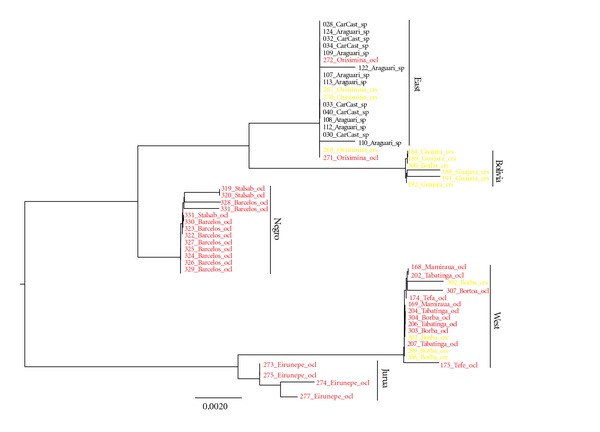
Maximum likelihood phylogenetic hypothesis (−ln⁡ = 1166.583) of relationships of individuals of *Astronotus* sampled throughout Brazilian Amazônia based on the mtDNA COI barcode region. The topology is significantly different (*P* = 0.003) from constrained topology enforcing monophyly of *A. ocellatus* and *A. crassipinnis*. red—*A. ocellatus* (ocelli present); yellow—*A. crassipinnis* (ocelli absent); black—unknown.

Using the ABGD software, we were able to infer five clades potentially representing species. Minimal divergence between these clades is 0.9% (Table 2). Individuals from all localities but Borba, a locality in the lower Madeira River, belong to just one clade. In the case of Borba, one individual is part of a clade that otherwise has a distribution in the Bolivian basin (upper Madeira River), while the remaining individuals are members of a clade found in the western Amazon basin. All five groups, with the exception of the Jurua group, are supported by at least one molecular synapomorphy (Table 3). For the sake of convenience, these clades will be referred to as East, Bolivia, Negro, West, and Jurua groups (Figure 3).

**Figure 4:**
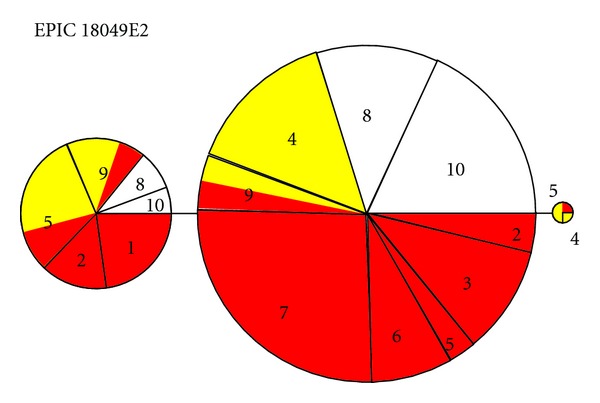
Haplotype network of the E0PIC 18049E2 nDNA region. Colors correspond to phenotypes: red—*A. ocellatus* (ocelli present); yellow—*A. crassipinnis* (ocelli absent); white—unknown. Numbers correspond to sampling localities: (1) Tabatinga; (2) Mamirauá; (3) Juruá; (4) Guajará Mirim; (5) Borba; (6) Santa Isabel; (7) Barcelos; (8) Careiro Castanho; (9) Oriximiná; (10) Araguari.

**Figure 5:**
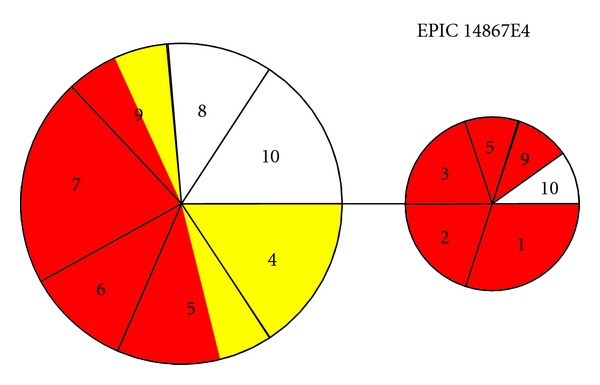
Haplotype network of the EPIC 14867E4 nDNA region. Colors correspond to phenotypes: red—*A. ocellatus* (ocelli present); yellow—*A. crassipinnis* (ocelli absent); white—unknown. Numbers correspond to sampling localities: (1) Tabatinga; (2) Mamirauá; (3) Juruá; (4) Guajará Mirim; (5) Borba; (6) Santa Isabel; (7) Barcelos; (8) Careiro Castanho; (9) Oriximiná; (10) Araguari.

The 18049E2 nDNA gene was represented by three haplotypes (Figure 4), with the most common haplotype being present in all localities but Tabatinga-western-most locality of the West clade, the second most common haplotype not occurring in the Negro River and upper Madeira River, corresponding to the Negro and Bolivia groups, and the third haplotype being restricted to the upper Madeira River—Bolivia group. The 14867E4 nDNA gene was represented by only two haplotypes (Figure 5), one common haplotype not found in western localities corresponding to the West and Jurua groups and another restricted to the central Amazonian localities. Both nDNA gene regions show strong structuring, that is, alleles are not randomly distributed among the five groups identified in ABGD analysis. Analysis of molecular variance of the 18049E2 nDNA gene was significant (*F*_ST_ = 0.4163, *P* < 0.001) as was that of the 14867E4 nDNA gene (*F*_ST_ = 0.8099, *P* < 0.001).

Ocelli were not phylogenetically clustered (Figure 3). A constrained topology where individuals with and without ocelli were forced into reciprocal monophyly, that is an explicit phylogenetic test of the usefulness of the presence/absence of ocelli as a diagnostic character, resulted in a significantly less likely topology (*P* = 0.003) and thus a rejection of the null hypothesis. However, analyses in CAPER indicated that ocelli were not distributed randomly across the ML topology (Fritz and Purvis' *D* = 0.3862, *P* < 0.001) but also were not clumped (*P* = 0.021).

## 4. Discussion

DNA barcode analyses revealed five, largely geographically restricted clades. Each clade with the exception of the Jurua group was supported by at least one molecular synapomorphy in the mtDNA dataset. While having less phylogenetic information, patterns of geographic distribution of nuclear DNA haplotype distribution did not contradict the mtDNA results and supported certain phylogeographic divisions observed in the mtDNA phylogeny. The Bolivia group had a private allele of the 18049E2 nDNA gene, while the second most common haplotype of this gene was absent in the Bolivia and Negro groups. Of the two 14867E4 nDNA alleles, the more common allele do not occur in the West and Jurua groups, while the rarer allele occurred infrequently in the group East.

The five groups predicted with Automatic Barcode Gap Discovery (ABGD) [26] and supported by the analyses of nuclear DNA loci can be taken as a first set of species hypotheses that need to be tested with other data. The algorithm is based on the statistical properties of the coalescent, and baring recent radiations, will identify evolutionary entities compatible with the coalescent. Other methods of identifying species from DNA barcode data are generally subjective or not generalizable across a broad range of organisms. The commonly used criterion of delimiting species such as the 3% interspecific divergence criterion, DNA barcodes differing by more than 3% belonging to different taxa [40], or the 10x rule, interspecific divergences that are 10x or larger than intraspecific divergences [41], fails to generalize for a number of taxonomic groups (e.g., [15, 42, 43]). Similarly, the interspecific and intraspecific divergences often overlap among closely related taxa (e.g., [15, 44–46]).

While it is clear that clades identified by ABGD [26] as potential species are geographically structured, the same cannot be said of the presence/absence of ocelli. Ocelli are not randomly distributed on the mtDNA phylogeny nor the nDNA haplotype networks; however, they also do not form monophyletic groups. Individuals of the Bolivia group do not have dorsal ocelli, while dorsal ocelli characterize all individuals of the Negro and Jurua groups. With the exception of individuals from the Borba locality, all other individuals pertaining to the group West are also characterized by the presence of ocelli. The group East is, on the other hand, characterized by a mix of individuals exhibiting both phenotypes (Figures 2 and 3). It should be noted that the Borba locality in the lower Madeira River is geographically intermediate between the Bolivia and the East groups. Thus, while some groups are monomorphic with respect to the presence/absence of ocelli, this character is not diagnostic and cannot be used to delimit species. Thus, currently, there are no morphological characters that can be used to diagnose and delimit species of *Astronotus*. On the other hand, ocelli are not randomly distributed throughout the phylogeny and do retain some phylogenetic information. In effect, specimens sampled from the vicinity of the main stream of the Amazon River (groups East and West) show both phenotypes, while specimens sampled from major affluents show either one or the other phenotype.

Broadly, however, the biodiversity patterns observed in the genus *Astronotus* are consistent with Kullander's [1] analysis. The group Bolivia is likely to be *Astronotus crassipinnis,* and one of its characteristics is lack of dorsal ocelli. What is currently considered *Astronotus ocellatus* harbors multiple species, a possibility also raised by Kullander, and while not diagnostic, specimens in the western Amazon basin have ocellated dorsal fin. Additional potential species currently subsumed under *A. ocellatus* include the groups from Jurua and Negro Rivers and from the central and eastern Amazon (group East).

The strong phylogeographic structure and the discovery of potentially new species of *Astronotus* are not necessarily surprising. *Astronotus* species are sedentary and territorial, have low power of dispersion, and therefore are likely to be influenced by climatic and geomorphological events. Perhaps the most interesting observation is that the division between the group East and West (not considering the Borba locality) parallels the division between the cichlid fishes *Symphysodon* sp. 2 (phenotype blue) and *Symphysodon tarzoo* (phenotype green) [16, 17, 47]. Also intriguing is that all but one specimen from the Borba locality in the lower Madeira River share haplotypes with the group West, which again parallels haplotype sharing between lower Madeira River and western Amazon observed by Ready et al. [47] in *Symphysodon*. The differentiation of the Bolivia group from all other *Astronotus* is potentially explained by the presence of the series of rapids on the Madeira River. These series of rapids are thought to delimit the geographic distributions of such diverse taxa as *Inia geoffrensis* and *I. boliviensis* [48, 49], *Cichla monoculus* and *C. pleiozona* [50], or they act as barriers, restricting gene flow in *Colossoma macropomum* [13] and *Podocnemis expansa* [51]. The physiochemical composition of the Negro River has also been suggested to act as a barrier between and within species [16, 17, 52, 53]. The patterns observed in *Astronotus* are likely to be general, implying that multiple additional species in broadly distributed Amazonian taxa are almost inevitably to be discovered.

## Figures and Tables

**Table 1:** Number of *Astronotus* specimens sampled at each site. We have no information about the phenotype (*Astronotus ocellatus*/*Astronotus crassipinnis*, presence/absence of dorsal ocelli, resp.) for specimens identified as *Astronotus* sp., but, in each of the Careiro do Castanho and Araguari River localities, both species of *Astronotus* occurred, were sampled, and were included in the analyses.

Localities	Specimen identification			All
	*A. crassipinnis*	*A. ocellatus*	*Astronotus *sp.
Tabatinga		4		4
Tefé/Mamirauá		4		4
Eirunepé		4		4
Guajará-Mirim	5			5
Borba	5	3		8
Barcelos		10		10
Sta Isabel do rio Negro		3		3
Careiro do Castanho			6	6
Oriximiná	3	2		5
Araguari river			8	8

Total	13	30	14	57

**Table 2:** Mean intra- and interspecific distances and their standard errors estimated between COI haplotypes using the Jukes Cantor model of molecular evolution [24]. Hypothetical species were inferred using the ABGD [26] algorithm.

Average divergence between groups (below diagonal), and associated standard errors (above diagonal)					
	East	West	Bolivia	Jurua	Negro
East		0.56%	0.36%	0.55%	0.36%
West	2.17%		0.55%	0.33%	0.57%
Bolivia	0.98%	2.20%		0.57%	0.42%
Jurua	2.08%	0.86%	2.42%		0.49%
Negro	0.97%	2.20%	1.31%	1.80%	

Average divergence within groups (left column), and standard errors (right column)					

East	0.03%	0.02%			
West	0.06%	0.03%			
Bolivia	0.10%	0.07%			
Jurua	0.13%	0.12%			
Negro	0.09%	0.05%			

**Table 3:** Matrix of molecular synapomorphies of the hypothetical species inferred using the ABGD [26] algorithm. Molecular synapomorphies are in bold. Column numbers indicate position within the sequenced COI fragment.

	89	98	131	143	152	209	215	227	236	248	260	305	443	447	464	539	578	590	596	662
East	G	C	G	G	T	T	C	A	A	T	T	C	T	C	A	A	**A**	T	C
Bolivia	G	C	G	A	T	T	C	A	A	T	C	C	T	C	A	**G**	G	T	**A**
Negro	**A**	C	G	G	T	T	C	G	A	C	C	T	T	C	A	A	G	T	C
West	G	T	**T**	A	C	C	A	A	G	T	C	T	C	**T**	G	A	G	A	C
Jurua	G	T	G	A	C	C	A	G	G	C	T	T	C	C	G	A	G	A	C
